# A kinematics-based model for the settling of gravity-driven arbitrary-shaped particles on a surface

**DOI:** 10.1371/journal.pone.0243716

**Published:** 2021-02-09

**Authors:** Mohsen Daghooghi, Iman Borazjani

**Affiliations:** 1 College of Science and Engineering, University of Houston-Clear Lake, Houston, TX, United States of America; 2 J. Mike Walker ’66 Department of Mechanical Engineering, Texas A&M University, College Station, TX, United States of America; Central State University & Ohio University, UNITED STATES

## Abstract

A discrete model is proposed for settling of an arbitrary-shaped particle onto a flat surface under the gravitational field. In this method, the particle dynamics is calculated such that (a) the particle does not create an overlap with the wall and (b) reaches a realistic equilibrium state, which are not guaranteed in the conventional discrete element methods that add a repulsive force (torque) based on the amount of overlap between the particle and the wall. Instead, upon the detection of collision, the particle’s kinematics is modified depending on the type of contact, i.e., point, line, and surface types, by assuming the contact point/line as the instantaneous center/line of rotation for calculating the rigid body dynamics. Two different stability conditions are implemented by comparing the location of the projection of the center of mass on the wall along gravity direction against the contact points to identify the equilibrium (stable) state on the wall for particles with multiple contact points. A variety of simulations are presented, including smooth surface particles (ellipsoids), regular particles with sharp edges (cylinders and pyramids) and irregular-shaped particles, to show that the method can provide the analytically-known equilibrium state.

## 1 Introduction

Settling of particles on a surface is an important phenomenon in natural and industrial systems such as dam-break collapse [1], particles on a vibrating plate [2] and granular flow on an inclined surface [3], among others. Particles in such systems have various shapes and sizes [4], but it is estimated that at least 70% of the raw materials consist of non-spherical particles [5]. The shape of particles cannot be simply approximated by an equivalent sphere because the dynamics of a single particle [6] or an entire particle system [7] of non-spherical shapes is different than those of spherical ones. This is true for settling behavior as well because there are various scenarios for geometrical contact between non-spherical particles and walls [5], in contrast to spherical ones. In fact, finding the final orientation (resting condition) for spherical particles over a surface is straightforward (rotation does not change the orientation of a sphere) while for non-spherical particles is particularly challenging.

One of the most successful numerical techniques to model particulate system is the discrete element method (DEM) [8]. In discrete models, a finite number of discrete particles (depending on computational resource and technique) are considered [9]. These particles can interact by means of contact and non-contact forces, and every particle, which can move translationally and rotationally, is described by Newton’s equations of motion and Euler’s equations of rigid body rotation [10, 11]. The gravitational force acts as a field force on particles in all discrete models [12] and is one prime cause of motion in various applications such as packing [13], particle sedimentation [14] and fluidization [15], when particles settle inside a container [16] or a hopper [17]. The translational motion due to gravitational force continues until particles reach the bottom of a container and collision occurs between the particle and the container’s surface. At this point, shape and surface characteristics of the particle and the wall determine the transient motion (usually of a rotation type) toward the final orientation of the particle with respect to the bottom surface, i.e., the equilibrium state. Collisions in discrete models are handled by calculating a contact force (moment) such that overlapping between particle (or between a particle and a wall) is avoided [12, 14]. However, the contact force (moment) is typically only applied when the particle and wall overlap (after actual collision) and it does not guarantee an equilibrium state which is physical. The method proposed in this work guarantees a physical equilibrium state for different non-spherical geometries.

A particle may rebound after collision with a wall if it has enough inertia, high coefficient of restitution, and high Stokes number [18] and the kinematics of particles can be obtained using impulse equations [19]. In such cases, auxiliary equations (coefficient of restitution and friction coefficient) along with some simplifying assumptions are needed to solve the problem [20–23]. Even if a rebound occurs, a fraction of the incoming kinetic energy of the particle is dissipated leading to a less energetic upcoming collision. This scenario could continue until the normal velocity (with respect to the plane surface) falls below a critical value for which rebound will not occur. This critical velocity depends upon the coefficient of restitution, adhesion energy, and geometry of the particle [18]. In other words, when a particle falls on a surface in a gravity-driven field, regardless of the number of rebounds (if any), there would be a final phase of the collision, in which no rebound would occur and the particle’s kinematics reaches a stable (equilibrium) state. This equilibrium state of particles in the final phase of collision, which is usually neglected in DEM, is the main focus of this work. In fact, to focus the problem on the equilibrium state, only the final phase of the collision in which no rebound occurs is considered in this work. Nevertheless, this method can be added to any previously developed particle-wall collision model (e.g. [22]), which models rebound from the surface, to numerically simulate a complete collision phenomenon including rebound(s) from the surface and a stable orientation on the surface.

This paper is organized as follows: in *Materials and methods* section, numerical schemes for particle representation, collision detection, different types of contact between a particle and a wall, equations of motion upon the collision, and stability conditions to detect the equilibrium state are presented and explained. In *Results and discussion* section, the framework is tested for a triaxial ellipsoid (an example for a smooth surface particle), a cylinder, a tetrahedron, and an irregular highly jagged particle (non-smooth particles) and results are qualitatively validated and discussed. Finally, our findings of the present framework as well as plans for future works are summarized in *Conclusion* section.

## 2 Materials and methods

The simulations presented in this paper are based on tracking the particle’s orientation during the wall collision. The fluid forces on the particle are neglected at this stage. Moreover, only smooth flat walls are considered. The change of translational and angular velocities of the particle during the wall collision process is calculated based on the Newton’s equations of motion and Euler’s equations of rigid body rotation.

**Fig 1:**
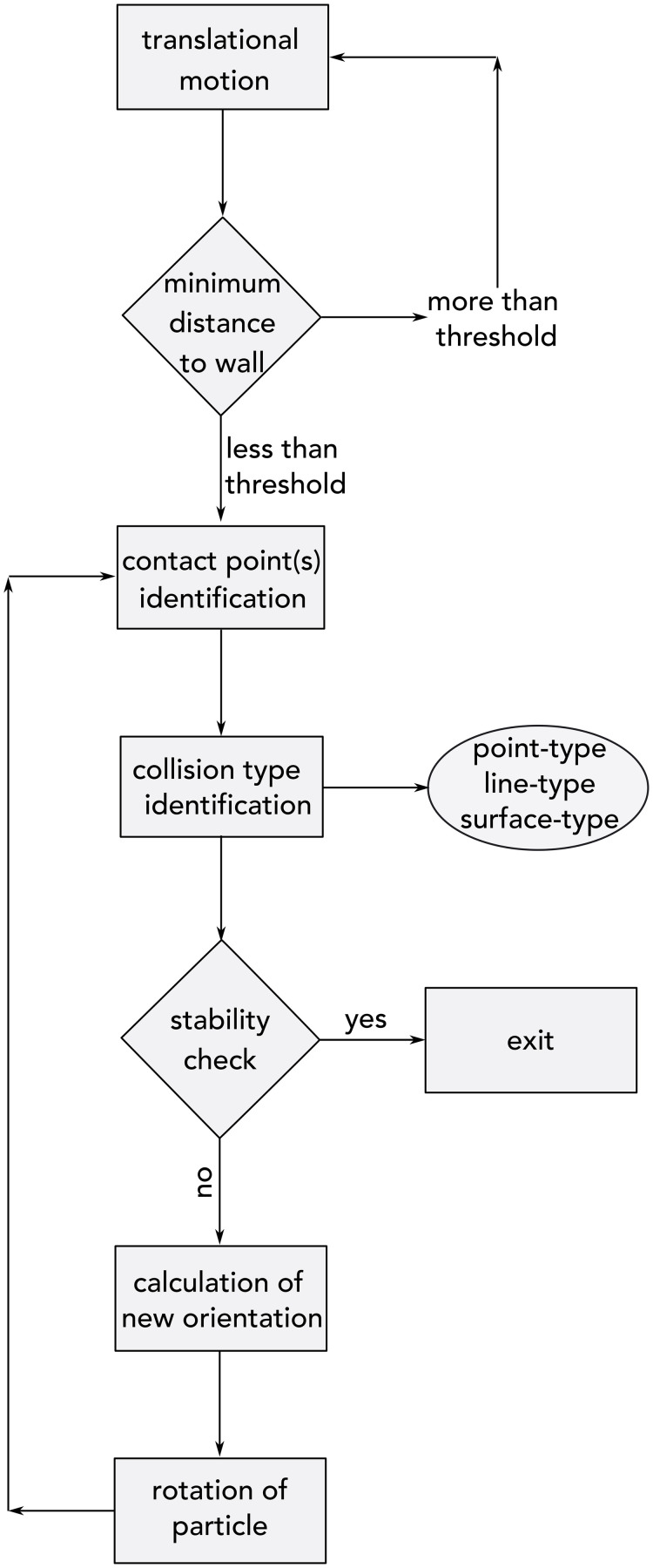
The flowchart shows essential steps in the wall collision treatment. The particle translates downward under the effect of the gravitational field and its distance with respect to the wall surface is measured. The collision between the particle and the wall is detected when the minimum distance becomes less than a critical threshold. All contact points are identified and depending on the number and distribution of contact points, the type of contact is distinguished. Two possible scenarios are checked to determine weather or not the particle has reached its equilibrium state. If the particle is not stable kinematics and orientation for the next time step is calculated and the particle is rotated according to its new orientation.

A brief overview of the framework can be seen in the flowchart of Fig 1 while details of each step will be explained in upcoming subsections. The particle translates downward under the effect of the gravitational field and its distance with respect to the wall surface is measured. The collision between the particle and the wall is detected when the minimum distance becomes less than a critical threshold and then all contact points are identified. Depending on the number and distribution of contact points, the type of contact is distinguished—see Fig 3. Then two possible scenarios are checked to determine weather or not the particle has reached its equilibrium state. If the particle is not stable, kinematics and orientation for the next time step is calculated and the particle is rotated to its new orientation.

### 2.1 Particle representation and collision detection

There are different methods to represent the particles, e.g., the surface discretization with different polygon/polyhedral elements [24], continuous functions to represent smooth non-spherical shapes (such as ellipses [25], super-quadratic curves [26], ellipsoids [27, 28], and 3D super-quadratic curves [29–31]), and discrete functions in which the shape is represented by an array of nodes with some specific associated information providing an ordered set of points [32, 33]. To handle arbitrary-shaped particles, the particles are discretized with triangular elements here. This enables us to represent different particles from smooth to irregular ones.

**Fig 2:**
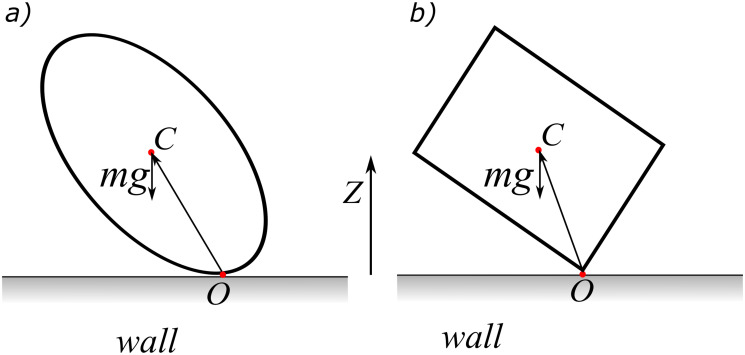
Kinematics during the collision depends on the shape of a particle. Shapes of particles categorized into two groups (a) strictly convex objects (curvature > 0) which smoothly oscillate to reach the stable position, and (b) objects with concave (curvature < 0) or non-smooth/flat (curvature = 0) surfaces which suddenly reach the stable position.

In our simulations, the particle moves under the gravitational field towards the wall. The first step in our methodology is to find when the collision occurs (Fig 1). The collision process starts when the minimum distance between the wall and the particle is smaller than a predefined threshold *ϵ*. This arbitrary threshold can be accounted for the surface roughness and in our simulations is set to be equal to *ϵ* = 0.05*L* where *L* is characteristic length of the particle (smaller values of *ϵ* have been successfully tested as well). Let the surface of the particle be discretized with an unstructured mesh consisting of a set of triangles and form a list of material points *k* = 1, *N* where *N* is the total number of material points for the particle. The minimum distance between the wall and the particle, *d* is defined as the closest distance of any material point on the surface of the particle with respect to the wall. Assuming *z* = 0 is the surface wall equation and the particle is falling under the gravitational force along the *z*-direction (see Fig 2), the minimum distance is defined as:

$$\begin{array}{c} d=Min\{\|z^{k}\|,k=1...N\} \end{array}$$ (1)

where *z*^*k*^ is the *z*-component of the position vector of *k*-th material point on the surface of particle in the Cartesian coordinate system. The point or a set of points whose minimum distance relative to the wall is equal or less than the threshold value (*d* ≤ *ϵ*) is considered as the contact point(s) of collision.

The distance measurement described above is fairly straightforward to implement but it is quite costly with a computational cost that is *O*(*N*). To reduce the computational costs a bounding box strategy is used. This bounding box strategy is similar to the “Bounding box” to identify immersed nodes in CURVIB method [34]. For a particle, the bounding box is defined as a Cartesian box that contains all vertices describing the solid object’s surface, i.e., the size of the bounding box is defined as:

$$\begin{array}{c} \left[x_{min}-\epsilon ,x_{max}+\epsilon \right]\times \left[y_{min}-\epsilon ,y_{max}+\epsilon \right]\times \left[z_{min}-\epsilon ,z_{max}+\epsilon \right] \end{array}$$ (2)

where *x*_*min*_ = *Min*{*x*^*k*^, *k* = 1…*N*}, *x*_*max*_ = *Max*{*x*^*k*^, *k* = 1…*N*}, etc. If the bounding box of the particles does not overlap with the wall surface, the particle is not about to collide and consequently, we do not need to measure the exact relative distance. This step allows us to run the minimum distance algorithm only when the particle is close to the wall.

### 2.2 Identification of contact type

The ultimate position of an object when falls and collides with a stationary surface is a stable one, but the kinematics during the collision depends on its shape. Shapes of particles that affect the collision kinematics are categorized into two groups in this work (Fig 2). The first group belongs to objects which have surfaces with a strictly convex shape (curvature strictly larger than 0), e.g. an ellipsoid (Fig 2a). Any other shape with a concave (curvature < 0) or discontinuous curvature such as objects with edges or flat faces (curvature = 0), e.g., a cube, a pyramid, or C shaped, falls into the second group (Fig 2b). Reaching the equilibrium (stable) position for the first group (hereinafter referred to as smooth particles) is achieved smoothly through a continuous oscillatory rotation, whereas for the second group (hereinafter referred to as non-smooth particles) stability occurs at a sudden moment when a face (sometimes some edges or head points) of the object totally lays on the wall, i.e, discontinuous rotation.

For smooth particles, a single point of contact is present at every time instant but the location of this point (referred to as the instantaneous center of rotation) can change as the object rotates (rolls). In this type of rotation, center of mass (COM) oscillates around the center of rotation until the object lays down on the wall such that COM has the minimum vertical distance with respect to the wall regardless of the initial position of the particle when collision occurs, i,e, the potential energy reaches its minimum value. The number of oscillations and the overall collision time depends on the surface roughness and object’s material (damping coefficient) and will be discussed in upcoming sections.

**Fig 3:**
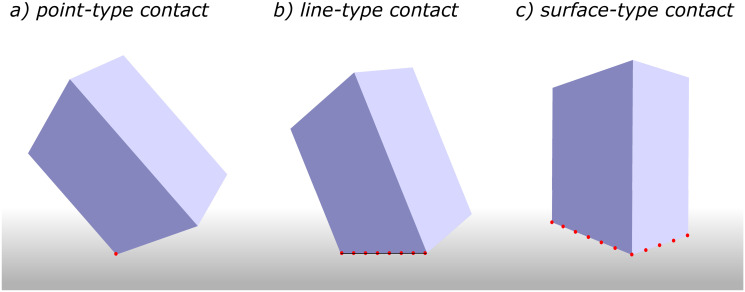
Different types of wall-particle contact. Various scenarios can happen when a particle collides with the wall: (collision points are shown as red dots) (a) point-type contact, (b) line-type contact, and (c) surface-type contact.

For non-smooth particles, however, the initial contact point does not usually change over time and the object just rotates about this point until it lays on a contact face (in some cases edges or heads). Various scenarios can happen when a non-smooth particle hits the wall. The initial collision can be either a point-type contact, a line-type contact, or a surface-type contact depending on the particle’s orientation and its shape (Fig 3).

#### 2.2.1 Point-type contact

When a sharp head of the particle’s surface collides with the wall, the initial contact point usually does not change and particle rotates about this point as this is an unstable position—see Fig 3a. In this type of contact, the rotation has three degrees of freedom (see section 2.3 for equations) and it continues until other points of particle’s surface reach the wall surface (within the threshold) and point-type contact turns into either a line-type contact or a surface-type contact. In the case of a very jagged surface, the particle could stay stable on a few contact points as will be discussed in the section 2.4.

#### 2.2.2 Line-type contact

This situation happens when a particle hits the wall on its edge—see Fig 3b. In this occasion, there are more than one contact point and all contact points forms a line, i.e. contact line. The particle rotates about this line until the situation would turn into a surface-type contact. In order to obtain this line numerically, first, all contact points are found and then the best fit (linear regression) for these points is obtained. Knowing the equation of the contact line, kinematics of rotation along this line are calculated. Note that in this situation, the rotation has only one degree of freedom.

#### 2.2.3 Surface-type contact

This situation happens when a particle hits the wall on its face—see Fig 3c. In this occasion, there are more than one contact point but these contact points are not located along a line and form a contact surface instead. This situation could be either stable or unstable depending on the particle’s orientation. The necessary condition for the stability of a particle is that the projection of COM on the wall lay inside the contact surface, as will be further discussed in the section 2.4. If the initial contact surface is not stable, particle rotates on one of its edges (surface-type contact turn into a line-type contact) and eventually lays on a stable face. Since in the surface-type contact there is no updated axis of rotation, the particle is rotated based on the axis of rotation from the previous time step. If the surface-type contact occurs at the beginning of rotation, the axis of rotation cannot be determined from the previous time step. In this case, the line of best fit (linear regression) is used as the axis of rotation.

The algorithm for detecting the contact type is provided in algorithm 1. The contact type for smooth particles is always point-type. For non-smooth particles if only one contact point is found, the collision is of point-type. Else, if there are more than one contact point and all contact points are aligned on a line the collision is line-type. Finally, if contact points don’t represent a geometric line, the collision is of surface-type. The line and surface types in the framework are distinguished by the magnitude of the residual sum of squares (RSS) of the linear regression technique used to find the best straight line that passes through contact points. If the RSS of the best line is less than a small threshold (0.1 in our work), contact points represent a line of contact, otherwise the collision is considered as a surface-type. Equations of motion for different types of contact are explained in the upcoming subsection.

### 2.3 Equations of motion after collision

To simulate the final phase of a collision in which no rebound occurs, it is assumed that the traslational motion stops and upon the collision the particle rotates about either a point or a line of contact. For smooth particles the rotation is always about a contact point, whereas for non-smooth particles both scenarios could occur, i.e., for point-type contact rotation is about a single point (similar to a smooth particle), and for line and surface types the particle is rotated about an axis of rotation. It should be noted that no sliding is considered in this work regardless of the type of contact.

**Algorithm 1: Identifying the type of contact between a particle and a wall**

All contact points are found;

*count* = number of contact points;

**if** *count* = *1* **then**

 contact is point-type;

**else**

 linear regression is used to find the best fit line through contact points;

 *RSS* = residual sum of squares;

 **if** *RSS* < 0.1 **then**

  contact is line-type;

 **else**

  contact is surface-type;

 **end**

**end**

**Fig 4:**
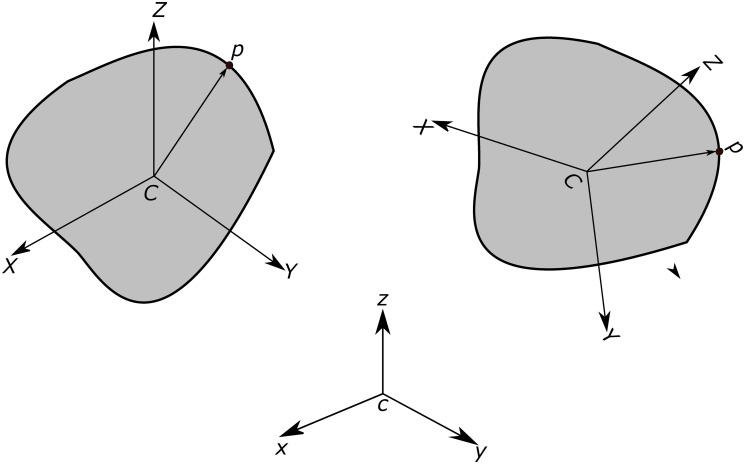
Orientation of the body with respect to the body space and the inertial coordinate systems. Body space coordinate system (*CXY* *Z*) moves and rotates with the same translational and angular velocity of the rigid body.

The shape of the rigid body is defined in terms of a references coordinate system attached to the body (shown as *CXYZ* in Fig 4) at the COM (choice of this point is arbitrary). This coordinate system is referred to as *body space*. As it is depicted in the figure, this coordinate system moves and rotates with the same translational and angular velocity of the rigid body. Therefore, the position vector of any material point *p* on the body’s surface remains unchanged overtime in this reference frame. Having the geometric description of the rigid body in the body space, we use rotation matrix R(*t*) to transform the body-space description into the inertial space *cxyz* description and find the instantaneous position vector of point *p* in the inertial coordinate system as:

$$r^{p}\left(t\right)=R\left(t\right){ℜ}^{p}+r_{c}\left(t\right)$$ (3)

where ***r***_*c*_(*t*) is the position vector of the particle’s COM and ℜ^*p*^ is the position vector of point *p* in the body space. Therefore, to fully define the orientation of the body, the rotation matrix and the vector position of COM need to be calculated at each specific time.

#### 2.3.1 Rotation about a contact point

When the contact between the particle and the wall is of point-type (either smooth or non-smooth surfaces), the particle rotates about the contact point (denoted by *O* in Fig 2) until it reaches to its final position. The total external torque with respect to the contact point *O* consists of the moment of the object’s weight with respect to point *O* and a damping (friction) torque as:

$$\sum M_{O}\left(t\right)=r_{OC}\times mg-C\omega$$ (4)

where ***r***_*OC*_ is the position vector of COM with respect to the collision point, *m* the mass, ***g*** the gravitational acceleration vector, C the damping coefficient, and ***ω*** the angular velocity of the object. Knowing the total external torque exerted on the body we can compute angular momentum of the body by integrating the following equation:

$$\begin{array}{c} \frac{dL_{O}\left(t\right)}{dt}=M_{O}\left(t\right) \end{array}$$ (5)

where ***L***_*O*_ is angular momentum of the body with respect to the contact point defined as:

$$\begin{array}{c} L_{O}\left(t\right)=I_{O}\left(t\right)\omega \left(t\right) \end{array}$$ (6)

where I_*O*_(*t*) is the instantaneous moment of inertia and can be calculated in terms of the constant-value moment of inertia in the body reference frame I^*B*^, the rotation matrix, and position vector of COM with respect to the collision point as:

$$\begin{array}{c} I_{O}\left(t\right)=R\left(t\right)I^{B}R^{T}\left(t\right)+m[\|r_{OC}{\|}^{2}E_{3}-r_{OC}r_{OC}^{T}] \end{array}$$ (7)

where ‖ • ‖ and •^*T*^ denote *L*^2^ norm and transpose operations, respectively and E_3_ is the 3 × 3 identity matrix.

The last equation to close the system of differential equation and fully determine the dynamic of rigid body rotation is time evolution of the rotation matrix due to the angular velocity of the body. However, in any numerical scheme for time integration of the rotational matrix some computational error is unavoidable. This error will be accumulated over time resulting in a skewing effect on the shape of the object [35]. In order to circumvent this problem and following our previous method [36], a quaternion vector is used instead of a rotational matrix to represent the orientation of the body in 3-D space [37]. Quaternions use four parameters to describe the three degrees of freedom of any arbitrary rotation. The advantage of implementing the quaternion method is that by re-normalizing the value of the quaternion to unit length, the numerical drift that is caused during the numerical integration can be eliminated [35]. Because of this property, it is desirable to represent the orientation of a body directly as a unit quaternion ***q***(*t*). Since we still need the rotation matrix to calculate the moment of inertia (Eq 7) and position vector (Eq 3), R(*t*) is computed as an auxiliary variable from the quaternion vector **q**(*t*) = [*q*_1_ *q*_2_ *q*_3_ *q*_4_]^*T*^ as follows:

$$\begin{array}{c} R=[\begin{array}{ccc} 1-2q_{2}^{2}-2q_{3}^{2} & 2(q_{1}q_{2}-q_{0}q_{3}) & 2(q_{1}q_{3}+q_{0}q_{2})\\ 2(q_{1}q_{2}+q_{0}q_{3}) & 1-2q_{1}^{2}-2q_{3}^{2} & 2(q_{2}q_{3}-q_{0}q_{1})\\ 2(q_{1}q_{3}-q_{0}q_{2}) & 2(q_{2}q_{3}+q_{0}q_{1}) & 1-2q_{1}^{2}-2q_{2}^{2} \end{array}] \end{array}$$ (8)

Finally, the time evolution of the quaternion vector is used to update the particle’s orientation during the simulation according to:

$$\begin{array}{c} \frac{dq(t)}{dt}=\frac{1}{2}q\left(t\right)\times \omega =\frac{1}{2}[\begin{array}{cccc} 0 & -\omega_{1} & -\omega_{2} & -\omega_{3}\\ \omega_{1} & 0 & -\omega_{3} & \omega_{2}\\ \omega_{2} & \omega_{3} & 0 & -\omega_{1}\\ \omega_{3} & -\omega_{2} & \omega_{1} & 0 \end{array}][\begin{array}{c} q_{1}\\ q_{2}\\ q_{3}\\ q_{4} \end{array}] \end{array}$$ (9)

Eqs 5 to 9 are discretized in time using explicit integration scheme and solved iteratively for all the unknown variables of the particle’s kinematics (***ω***, R, **q**) as follows:

1. The total torque exerted on the particle for the current time step *n* + 1 is calculated explicitly based on the known angular velocity and vector position of COM from the previous time step *n* (see Eq 4).
  $$M^{n+1}=M\left(\omega^{n},q^{n}\right)$$ (10)
2. Eq 5 is integrated explicitly (trapezoidal rule) to obtain angular momentum for time step *n* + 1.
  $$L^{n+1}=L^{n}+\Delta tM^{n+1}$$ (11)
3. To obtain orientation and angular velocity of the body for the next time step we have to solve coupled nonlinear Eqs 6 to 9 iterating over pseudo-time step *l* as:
  $$\frac{q^{l+1}-q^{l}}{\Delta \tau }+\frac{q^{l}-q^{n}}{\Delta t}=\frac{1}{2}(\frac{1}{2}q^{l}\times \omega^{l}+\frac{1}{2}q^{n}\times \omega^{n})$$ (12)
  $$R^{l+1}=R(q^{l+1})$$ (13)
  $$I_{O}^{l+1}\left(t\right)=R^{l+1}I_{b}{R^{l+1}}^{T}+m[\|r_{OC}^{n}{\|}^{2}E_{3}-r_{OC}^{n}{r_{OC}^{n}}^{T}]$$ (14)
  $$\omega^{l+1}={\left(I^{l+1}\right)}^{-1}L^{n+1}$$ (15)
4. Check for convergence of the quaternion solution:
  $$\|q^{l+1}-q^{l}\|<\epsilon$$
  where *ϵ* is a preset convergence ratio set equal to *ϵ* = 10^−8^.
5. At the convergence we set:
  $$q^{n+1}=q^{l+1}$$ (16)
  $$R^{n+1}=R^{l+1}$$
  $$\omega^{n+1}=\omega^{l+1}$$

Upon the calculation of kinematics for the current time step *n* + 1, the orientation of particle is updated (according to Eq 3) by rotating it about the collision point.

#### 2.3.2 Rotation about a line of contact

Equations of rotational motion explained earlier are used for the rotation about a point for both smooth and non-smooth particles. For rotation about an axis of rotation which might occur during the rotation of non-smooth particles for line and surface types of contact, the unit vector of rotation axis ***e***_***r***_ is first calculated using linear regression technique through all contact points. Then the external torque is projected on this vector as:

$$\sum M_{r}\left(t\right)=(r_{OC}\times mg-C\omega ).e_{r}$$ (17)

Eqs 5 to 9 are integrated in time as explained earlier (for a rotation about the collision point) but this time the updated angular velocity is projected on the axis of rotation as:

$$\omega_{r}\left(t\right)=(\omega .e_{r})e_{r}$$ (18)

Consequently, the particle would be constrained to rotate about the collision line.

### 2.4 Stability condition

As mentioned earlier, in the final phase of collision a smooth particle goes through a continuous oscillatory rotation until it reaches its final position (minimum gravitational potential energy). For a non-smooth particle, there is a different scenario. During the rotation, there will be a unique orientation in which the particle is stable and the rotation suddenly stops. If this stable orientation is not detected, the particle would oscillate on a flat/concave surface, which is non-physical. Therefore, checking the stability of a non-smooth particle during the rotation is essential for realistic simulations.

**Fig 5:**
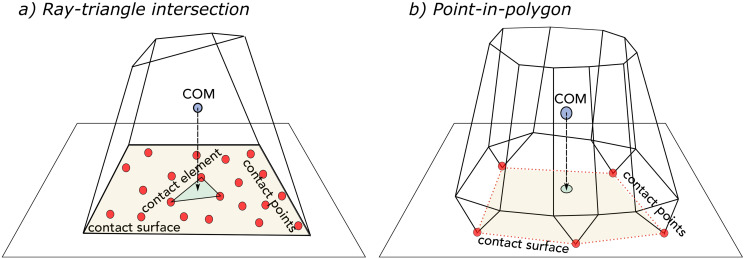
Sketch showing the ray-casting method. (a) In the ray-triangle scheme, a half ray shoots from the COM along the vertical direction. if this ray intersects with a contact element it means the COM projection on the wall lay inside the contact surface and the particle is stable. (b) In a point-in-polygon scheme, a polygon consisting of all contact points is considered. If the projection of particle’s COM on the wall is inside this polygon, the particle is considered stable.

**Fig 6:**
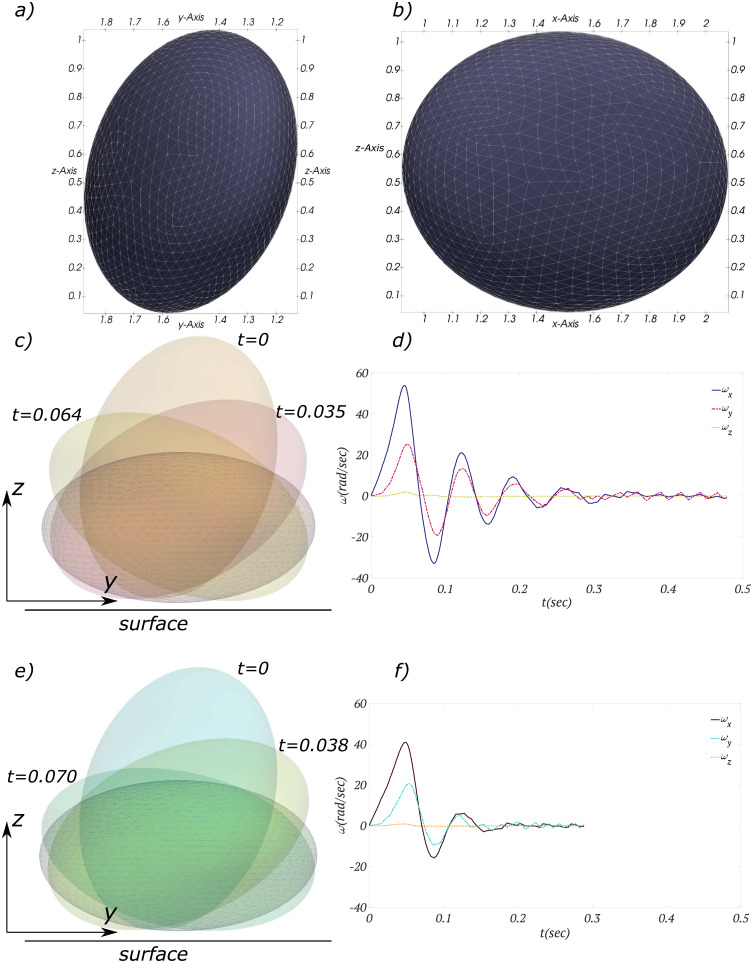
Settling of a triaxial ellipsoid on a flat surface. a) and b) Projections of the particle on Cartesian coordinate planes with correlated scales are shown for the initial orientation. c) and d) Angular velocity and time series of the particle with damping coefficient C=0.75 are shown. e) and f) Angular velocity and time series of the particle with damping coefficient C=1.5 are shown.

**Fig 7:**
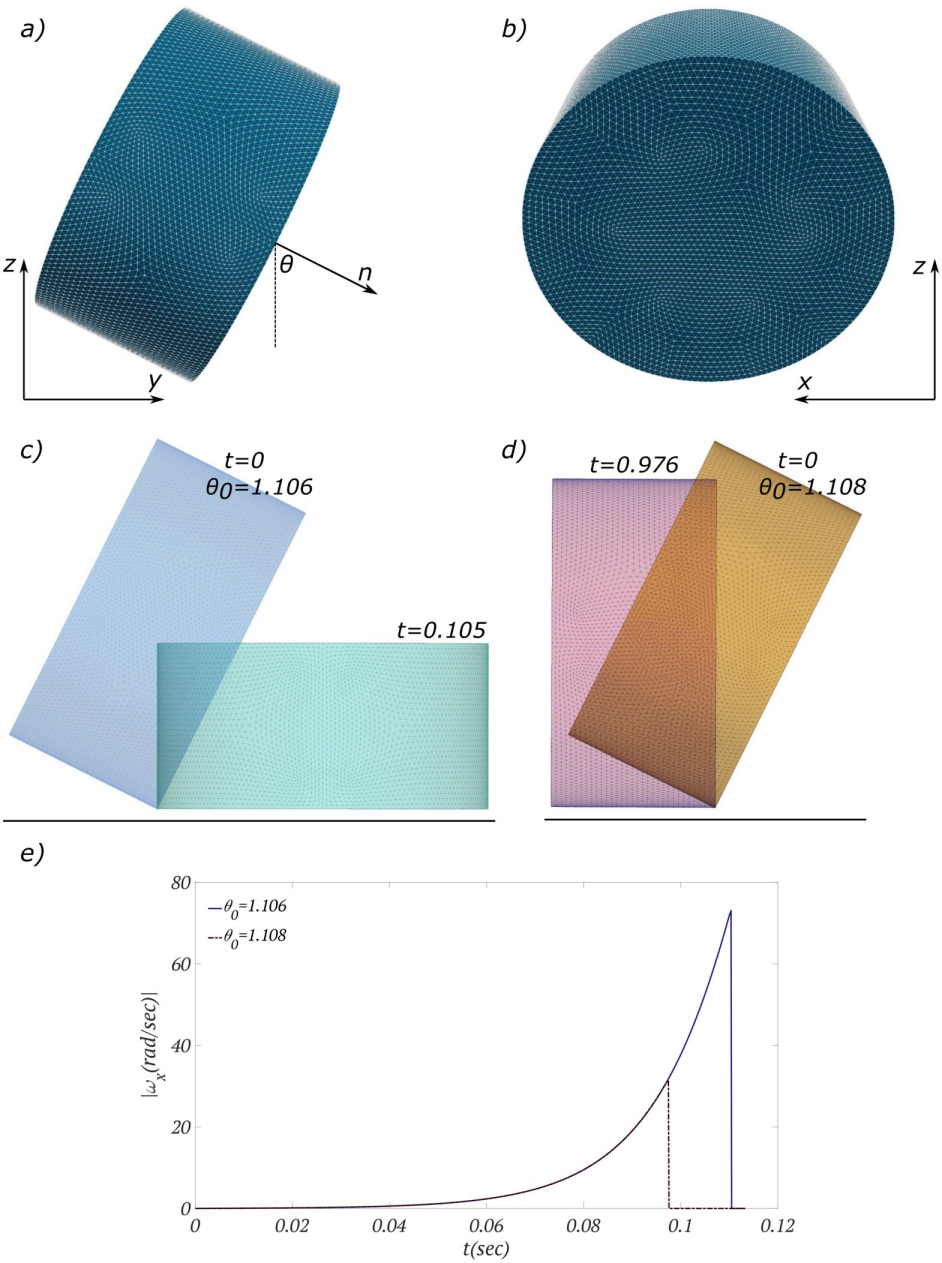
Settling of a cylinder on a flat surface. a) and b) Initial orientation of the particle with respect to an inertial frame of reference *cxyz* is shown. c) Axis of the cylinder is initially at *θ*_0_ = 0.106*rad* and it finally lays on its base surface. d) Axis of the cylinder is initially at *θ*_0_ = 0.108*rad* and it finally lays on its side surface. e) Angular velocity of both cases during the settling on the surface are compared.

**Fig 8:**
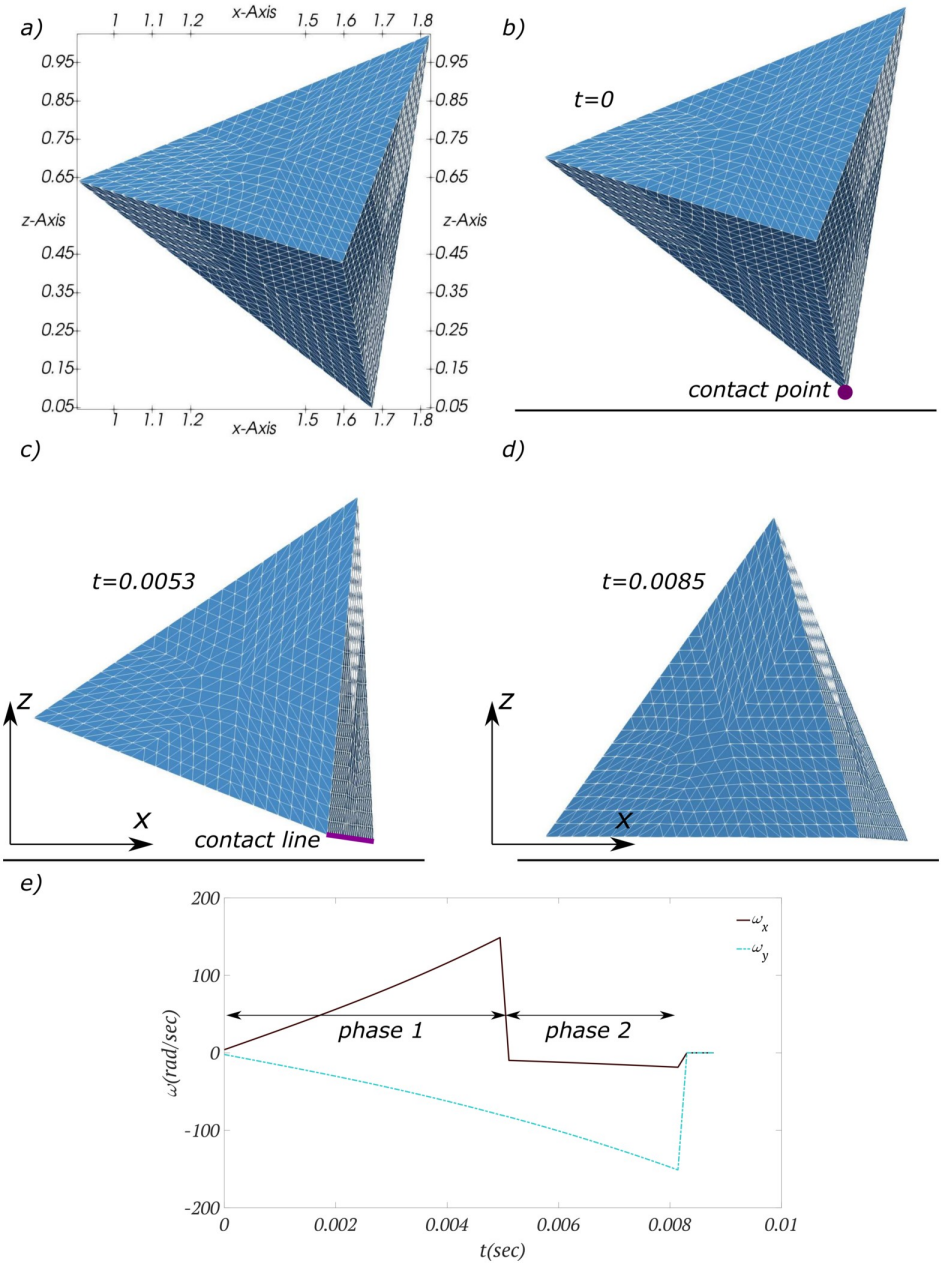
Settling of a regular tetrahedron on a flat surface. a) Projections of the particle on Cartesian coordinate planes with correlated scales are shown for the initial orientation. b) The particle collides with the wall with its head and a point-type collision occurs. c) Multiple contact points form a contact line. d) The pyramid lays on one of its flat surfaces at equilibrium. e) Two components of angular velocity are show. Sudden change in angular velocity is the result of the change in axis of rotation, i.e. transition from phase 1 to phase 2.

Two situations for the stability of a particle are monitored when it collides with a surface. In the first stability condition, which occurs more frequently, a contact surface consisting of all contact points is detected. Contact points on the contact surface belong to a face of the particle and, consequently, these material points belong to the triangular surface mesh on the particle surface (see Figs 6–8). If all vertices of a triangular mesh are within the collision threshold, i.e., all vertices are contact points, we call the mesh element a contact element (green triangle in Fig 5a). After all contact elements are detected, a ray-casting algorithm adopted from [38] is used to see if the projection of the particle’s COM intersects with any of contact elements. If a half ray shoots from the COM along the vertical direction intersects with a contact element that means the COM projection on the wall lays inside the contact surface and the particle is stable (see Fig 5a). The basic idea of this ray-triangle intersection method is that for a triangle defined by its three vertices $\left(v_{i}^{1},v_{i}^{2},v_{i}^{3}\right)\left(i=1,2,3\right)$, where $v_{i}^{j}$ is the *i*^*th*^ Cartesian coordinate of the *j*^*th*^ vertex, any point *T* = (*τ*_1_, *τ*_2_, *τ*_3_), where *τ*_*i*_ is the *i*^*th*^ Cartesian component of point *T*, within the triangle can be expressed as follows

$$\tau_{i}\left(a,b\right)=\left(1-a-b\right)v_{i}^{1}+av_{i}^{2}+bv_{i}^{3}$$ (19)

with *a* ≥ 0, *b* ≥ 0 and *a* + *b* ≤ 1. The ray shoots from COM and pointing downward can be expressed as Γ = (*C*_1_, *C*_2_, *C*_3_ − *δ*), where *C*_*i*_ is the *i*^*th*^ Cartesian component of COM and *δ* > 0. Finding the intersection between the ray and the triangle is equivalent to finding the solution to the following equation:

$$\Gamma_{i}\left(\delta \right)=\tau_{i}\left(a,b\right)$$ (20)

If the solution to Eq 20 satisfies *δ* > 0, *a* ≥ 0, *b* ≥ 0 and *a* + *b* ≤ 1, then the ray intersects with a triangle.

In the second stability condition, which only happens for highly jagged particle surfaces, very few collision points are found (Fig 5b). These points, contrary to the previous scenario, do not necessarily belong to a collision element, i.e., they may belong to surface elements that are not entirely lay on the wall surface. The number of these collision points for this type of surface are significantly less than the previous situation and are quite limited. After identifying all collision points, a polygon consisting all these points is considered and then we check to see if the projection of particle’s COM on the wall (shown as green dot in Fig 5b) is inside this polygon, which indicates the stability of the particle. This test is the classical problem of point-in-polygon problem in computational geometry. This problem is solved by the so-called ray-casting method [39], which requires the location of the polygon vertices (contact points) and the point (projection of COM on the wall) as inputs. Starting from the projection of particle’s COM on the wall (green dot in Fig 5b), a random half-infinite ray is casted and the number of intersections between the half ray and polygon edges (shown as dotted lines in Fig 5b) is counted. If the number of intersections is odd then the point is located inside the polygon, otherwise it is located outside. Note that, we automatically check both scenarios for the stability of a particle and only one is a sufficient condition to consider the particle stable.

## 3 Results and discussion

In this section, the method is validated and verified against analytical and benchmark solutions. It is shown that the method can simulate the final phase of a collision (without rebound) until the particle reaches a physical and realistic equilibrium (stable) position. Results for one smooth and three non-smooth particles are presented and discussed.

As it was mentioned in *Materials and methods*, for smooth particles the final stable state is reached smoothly through a continuous process in terms of kinematics (e.g. angular velocity) and there is always a single collision point. For a non-smooth particle, on the other hand, the final stable state is reached abruptly and the type of contact could change from a point to a line and finally to a surface type. A major difference in terms of implementing the governing equation for both surface types is the value of the damping coefficient in Eq 4. The value of the damping coefficient C depends on many factors such as surface roughness and object’s material. For particles with a continuous curvature (smooth) a non-zero positive value can be considered C>0, whereas for non-smooth particles, it is assumed that the particle does not oscillate around the contact point but simply rotates until it lays over one of its surfaces or some single contact points abruptly. This assumption is physically acceptable for objects whose density is significantly higher than air. To implement this scenario the damping coefficient for a non-smooth particle is set to be zero (C=0).

### 3.1 Smooth particles

The settling of a gravity-driven triaxial ellipsoid on a plane surface is simulated for two different values of damping coefficients (Fig 6)—see also the S1 Video. The triaxial ellipsoid has major diameters of (1.25, 1, 0.57)*cm* and density of 1.2*gr*/*cm*^3^ and it is initially rotated such that its orientation with respect to the inertial frame of reference *cxyz* (see Fig 4) agrees with what is shown in Fig 6a and 6b. The damping coefficient for two tested cases are chosen as $C=\left(0.75,1.5\right)gr.cm^{2}/sec$.

At the beginning of the collision procedure (*t* = 0 in Fig 6c and 6e), the particle’s COM has the highest elevation with respect to the surface and it is located at the right-hand side of the collision point. Therefore, the particle starts to rotate clockwise. This angular motion for the particle with the lower value of damping coefficient C=0.75 (shown in Fig 6c and 6d) is faster than the one with the higher value of C=1.5 (shown in Fig 6e and 6f). This can be easily verified by comparing amplitude of components of angular velocity *ω*_*x*_ and *ω*_*y*_ in Fig 6d and 6f. For instance, for the particle with a lower damping coefficient it takes *t* = 0.035*sec* to sweep *θ* = 0.65*rad* along *x*–*axis*, while for the particle with a higher damping coefficient the required time for the same swept angle is *t* = 0.039*sec*. The clockwise rotation continues until the particle’s COM reaches its second elevation peak. The elevation of this peak is inversely proportional to the damping coefficient. In other words, the particle with damping coefficient C=0.75 sweeps a maximum angle of *θ*_*max*_ = 1.79*rad* during the first oscillation, which takes *t* = 0.064*sec*, whereas for the particle with damping coefficient C=1.5 the required time for the maximum swept angle of *θ*_*max*_ = 1.55*rad* is *t* = 0.070*sec*. At this time instant, the particle’s COM is located at the left-hand side of the collision point and consequently, the particle starts to rotate counterclockwise. The counterclockwise rotation continues until the particle’s COM reaches to its third peak, which is lower than previous ones. This cycle continues and particle oscillates around the collision point until it reaches the final steady state position (Fig 6c and 6e) in which the particle’s COM has the minimum elevation, i.e., minimum gravitational potential energy. Fig 6d and 6f clearly show that the higher the value of damping coefficient, the sooner the particle reaches steady state with fewer oscillations around the contact point. This type of rotation with alternative signs and a damping amplitude qualitatively agrees with numerical results of Mohaghegh and Udaykumar [23] for a 2-D ellipse in a viscous fluid.

### 3.2 Non-smooth particles

Contrary to a smooth particle, the final equilibrium orientation for a non-smooth particle during the settling could depend on its initial orientation. For instance, Fig 7a and 7b show a cylindrical particle of density 0.96*gr*/*cm*^3^, height of *h* = 0.67*cm* and radius of *r* = 0.67*cm* with an axis oriented at angle *θ* with respect to *z*-direction. It can be analytically verified that the gravitational moment about the contact point (the first term on the right-hand-side of Eq 4) changes its sign at a critical angle of *θ*_*c*_ = *π*/2 − arctan(*h*/2*r*). In other words, for the cylinder shown in Fig 7, the gravitational moment about the *x*-axis is negative for *θ* < 1.107*rad* and positive for *θ* > 1.107*rad*, and *θ* = 1.107*rad* is the unsteady equilibrium position. Since the damping coefficient is set be zero for non-smooth particles, the sign of angular velocity and, consequently, the angle of rotation agrees with the sign of the gravitational moment. In order to numerically verify this result, we tested the cylindrical particle with two initial orientations: *θ*_0_ = 1.106*rad* (shown in Fig 7c) and *θ*_0_ = 1.108*rad* (shown in Fig 7d).

For both initial orientations the rotation starts from the vicinity of the unsteady equilibrium position *θ*_0_ = 1.107±0.001*rad* and in both cases, collision starts with a handful of adjacent contact points. The axis of rotation is consequently obtained by finding the best line that passes through collision points during the rotation as explained in section 2.2 (see *line-type contact*). As can be seen in Fig 7c and 7d, the axis of rotation remains along *x*-axis in both cases, where the cylinder initially starts from *θ*_0_ = 1.106 rotates clockwise (*ω*_*x*_ < 0), and the cylinder initially starts from *θ*_0_ = 1.108 rotates counterclockwise (*ω*_*x*_ > 0).

The absolute value of angular velocity for both cases are shown and compared in Fig 7e. Since the initial conditions in both cases are symmetric about the equilibrium position, the rotations are expected to be symmetric as well. This analytical answer is verified by our numerical results and as it can be clearly seen in the plot, the magnitude of angular velocities are equal (with opposite signs) at any given time instant. The rotation for the cylinder initially started from *θ*_0_ = 1.108 takes *t* = 0.976*sec* sweeping an angle of Δ*θ* = 0.463*rad*, whereas the other cylinder rotates for a longer time *t* = 1.105*sec* to sweep Δ*θ* = 1.106*rad*.

Upon reaching the equilibrium state, particle’s motion terminates abruptly (surface-type collision) where a line-type collision is followed by a surface-type collision—see also the S2 Video. One might argue that for the cylinder initially started from *θ*_0_ = 1.108, the final collision state should be of a line-type mathematically, but in real physical and computational situations, there would be a narrow strip contact surface when the cylinder become steady as shown in Fig 7d.

Another example that can show the transition from a point-type contact to a line-type contact before a final steady surface-type collision is shown in Fig 8. The regular tetrahedron in this simulation has a density of 1.2*gr*/*cm*^3^ and all four faces are equilateral triangles with edge length of 1*cm*. When the particle reaches the surface for the first time, it collides with the wall with its head, i.e., a point-type collision (Fig 8b). In this collision type, there is one constraint in the rotation as the contact point does not change during the rotation. The axis of rotation passes through this contact point and is perpendicular to the line that connects the contact point and the projection of COM on the wall surface. The triangular pyramid rotates due to its gravitational torque about this axis until other points located on one of its edges collide with the wall and a contact line is formed (Fig 8c). This time instant can be tracked on the plot of Fig 8e, which shows components of angular velocities, as the first discontinuity. The first phase of rotation from the appearance of contact point till the formation of contact line takes Δ*t* = 0.0053*sec* for this simulation.

When the contact line forms, the particle rotates about this line, i.e., contact line acts as the new axis of rotation (Fig 8c). The sudden change in the axis of rotation could result in a abrupt change in components of angular velocities. For instance, upon the transition from contact point to contact line (phase one to phase two of rotation), *ω*_*x*_ changes its sign from positive to negative in Fig 8e. The rotation in this phase continues until other points on the bottom face of the particle reach the threshold distance for the collision with the wall, i.e., a surface-type collision is detected. This time instant can be seen on the plot of Fig 8e as the second discontinuity. The second phase of rotation from the appearance of contact line until the formation of contact surface takes Δ*t* = 0.0032*sec* for this simulation.

The surface-type collision is immediately followed by the stable condition when the COM projection intersects with a contact element, i.e., Ray-triangle intersection stability condition (Fig 5a). In this final state, the pyramid is on one of its flat surfaces (Fig 8d). Three types of contact and transition from one to another can be better seen in the S3 Video.

**Fig 9:**
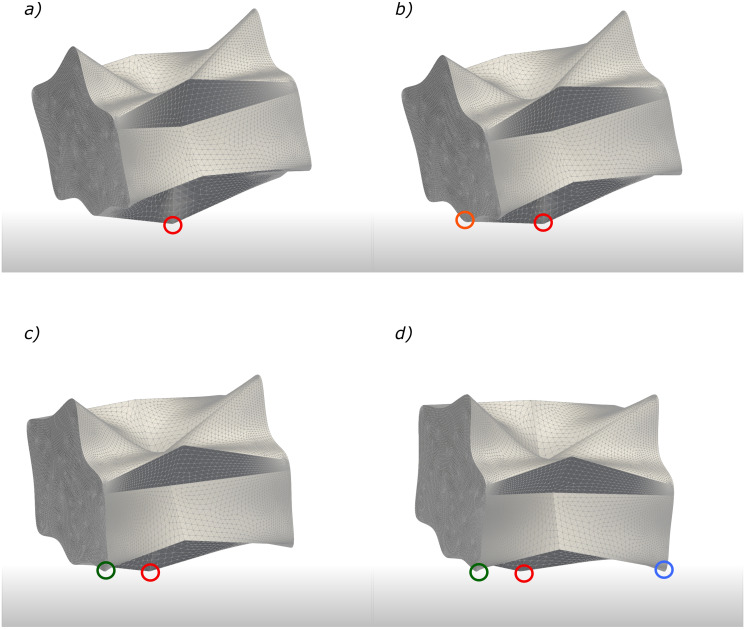
Time series of the collision procedure and resting of an irregular particle on a rigid wall is shown. For this type of particle shape in the shown orientation only a few collision points (indicated as color circles) are expected. a) The particle first hits the wall with a single sharp head and starts to rotate around this point. b) The rotation continues until the second collision point is found. From this time, the particle rotates along an axis, which passes through both collision points. c) After a bit rotation along the first axis, another collision point is found and replaces the second one. d) Finally, particle is rested on three collision points such that the projection of COM on the wall locates inside these points.

As one might expect and previously mentioned in section 2.4, some particles have highly jagged surfaces and do not have flat or smooth surfaces similar to what are shown in Figs 6–8. In order to show the capability of our framework to handle collision and stability of any arbitrary irregular-shaped particle, a highly skewed and jagged-shape particle is tested and the result is shown in Fig 9—see also the S4 Video. For this type of particle shape as shown in the figure, only a few contact points (indicated as color circles) can be expected depending on the initial orientation. Similar to previous tests, the particle first hits the wall with a single sharp head and starts to rotate around this point (red circle in Fig 9a). The rotation continues until the second contact point (orange circle in Fig 9b) is found. From this time, the particle rotates along an axis, which passes through both contact points. If there are more than two points, the best fit line technique would be used (see section 2.2). Similar to the rotation of a tetrahedron in Fig 8, for this particle, the axis of rotation can change as the surface is jagged and bumpy. This can be seen in Fig 9c where a new contact point (green circle in Fig 9c) is found after a bit of particle rotation about the first axis and replaces the previous contact point. Orientation of the axis of rotation changes at this moment, consequently. The shift of the rotation axis from the first one to the second one can be better seen in the S4 Video. This procedure is repeated until the particle rests stably on several collision points (Fig 9d) such that the projection of COM on the wall locates inside a polygon that passes through collision points (see Point-in-polyhedron stability condition and Fig 5b).

## 4 Conclusion

A discrete element algorithm is developed to resolve the settling of an arbitrary-shaped particle on a flat surface. In this method, the particle’s orientation is solved such that the particle reaches a realistic and physical equilibrium (stable) state, which may not be guaranteed by traditional DEM that add a contact (repulsive) force (torque) based on the amount of overlap. This is achieved by identifying the type of contact (section 2.2), i.e., point, line, surface; solving the rigid body dynamics based on the type of contact (section 2.3); and identifying the equilibrium (stable) state (section 2.4), i.e., stable on a surface or on a few contact points. The method has been validated for different geometries and shown to model the analytical stable state.

It should be noted that the current method does not consider rebound when particles fall on a surface. This is an acceptable physical assumption for several gravity-driven objects including particles with small normal velocity (low incoming kinetic energy) or those whose inertia is at the order of the viscous force exerted from the ambient fluid especially for non-smooth surfaces. Even in cases that a particle (usually smooth particles) bounces back from the surface, it loses some of its momentum (energy) during each collision and eventually goes through a collision without bouncing, i.e., final phase of collision. Therefore, this method can be integrated with other collision treatments, in which the rebound from the surface is also a part of a wall-particle interaction, e.g., non-zero coefficient of restitution and friction [22]. This will be carried out as part of a future work. In fact, this method can be implemented in any discrete element model to obtain a more accurate and realistic final equilibrium state of a particle on the wall. In addition, this numerical scheme will be coupled to our immersed boundary method [6, 36] to simulate sedimentation of rigid particles in a viscous fluid in the future.

## Supporting information

S1 Video Settling of a triaxial ellipsoid on a plane surface. (MOV)

S2 Video Settling of a cylinder on a plane surface. (AVI)

S3 Video Settling of a tetrahedral pyramid on a plane surface. (AVI)

S4 Video Settling of a irregular-shaped particle on a plane surface. (AVI)
